# KIFC1 promotes proliferation and pseudo-bipolar division of ESCC through the transportation of Aurora B kinase

**DOI:** 10.18632/aging.205203

**Published:** 2023-11-08

**Authors:** Bin Du, Lingyu Wei, Jia Wang, Yanyan Li, Jing Huo, Jinsheng Wang, Pu Wang

**Affiliations:** 1 Center of Healthy Aging, Changzhi Medical College, Changzhi 047500, China; 2 Department of Pathology, Affiliated HePing Hospital of Changzhi Medical College, Changzhi 047500, China; 3 Department of Pathology, The First Clinical College of Changzhi Medical College, Changzhi 047500, China; 4 Department of Biology, Changzhi Medical College, Changzhi 047500, China

**Keywords:** KIFC1, Aurora B, ESCC, proliferation, pseudo-bipolar division

## Abstract

Esophageal squamous cell carcinoma (ESCC) accounts for over 90% of total in China, and the five-year survival rate for patients is less than 30%. Accordingly, the identification of novel, effective early diagnosis markers and therapeutic targets for ESCC is of paramount importance. KIFC1 has been identified as highly expressed in several types of cancer, although its prognostic value is inconsistent, and no research has been conducted specifically on its effect on ESCC. To investigate the expression and function of KIFC1 in ESCC, we conducted immunohistochemical staining on 30 pairs of para-carcinoma tissue and cancerous tissues, revealing a significant increase in KIFC1 expression in ESCC tissues. Using siRNA to knock down KIFC1 significantly reduced the proliferation of EC109 ESCC cells both *in vitro* and *in vivo*. Bioinformatics analysis revealed a highly significant positive correlation between KIFC1 overexpression and signaling pathways associated with tumor proliferation pathways. In EC109 cells, overexpression of KIFC1 significantly increased the rate of centrosome amplification and the likelihood of pseudo-bipolar division. Furthermore, the expression of KIFC1 and the rate of centrosome amplification in ESCC tissues were also positively correlated. In order to explore the underline molecular mechanisms, we identified, through proteomics, that KIFC1 binds to the protein Aurora B. The knockdown of KIFC1 significantly reduced the distribution of Aurora B on the metaphase plate and substantially inhibited the phosphorylation of its classical substrate, Histone H3. In conclusion, these findings indicate the potential utility of KIFC1 as both a tumor marker and a promising target for therapeutic interventions.

## INTRODUCTION

Esophageal carcinoma (EC) is the eighth most prevalent and the sixth most deadly cancer globally [1]. EC is a heterogeneous disease which was categorized into two subtypes: esophageal adenocarcinoma (EAC) and esophageal squamous cell carcinoma (ESCC). Approximately 90–95% of the EC patients were diagnosed with ESCC in the Taihang mountains of north-central China [2]. Due to the limited clinical approaches for early diagnosis, ESCC tends to have a poor prognosis. The 5-year survival rate of EC ranges from 10% to 25% [3]. At present, despite the substantial advancements in neoadjuvant chemotherapy and surgical interventions, the prognosis of esophageal cancer remains unsatisfactory. Consequently, the exploration of novel early diagnosis marker and therapeutic targets carries significant importance.

KIFC1 (Kinesin Family Member C1) is a protein primarily known for its role within the molecular motors of cells. KIFC1 plays a pivotal role in executing numerous fundamental cellular operations, ranging from intracellular transportation to cell division. It is noteworthy that aberrations in the expression levels of KIFC1 have been demonstratively linked with progression in specific categories of cancer [4–6].

KIFC1 is highly expressed in a variety of tumors. Many studies suggest that KIFC1 could be used as a potential actionable biomarker of early-stage tumorigenesis and progression of high-risk lesions [7]. However, the protein expression of KIFC1 in some tumors was not significantly elevated from that in normal tissues. It was unsuitable for tumor marker, such as lung cancer, colon cancer, and cervical cancer [8]. However, within the context of esophageal cancer, the expression parameters of KIFC1 remain largely underexplored.

KIFC1 was associated with poor prognosis in several tumors. Targeting KIFC1 could significantly enhance the lethality of chemotherapeutic drugs for tumor cells [9], whose underlying mechanisms may involve centrosome de-cluster [10], energy metabolism [11] and endoplasmic reticulum dysfunction [5]. Existing data show that high expression of KIFC1 could support the proliferation of tumor cells and improve the cell’s ability to withstand centrosome amplification and multipolar division, thereby increasing the instability of the genetic material [12]. Mechanistically, KIFC1 was regulated by TCF-4, the critical regulator of the Wnt/β-catenin pathway, and promotes the transcription of HMGA1 [13]. Deletion of KIFC1 results in the degradation of lamin B and A/C, defective spindle assembly, formation of micronuclei, as well as loss of chromosomes during mitosis [10]. Coincidentally, KIFC1 was shown to promote the proliferation of tumor cells by regulating AKT, CENPE, ZWINT, and other pathways [14–16]. In liver cancer, KIFC1 could enhance the transcriptional activity of HMGA1, thereby accelerating the expression of downstream genes [13].

Indeed, research has revealed that KIFC1 can be phosphorylated by ATM/ATR and robustly uphold spindle stability when cells are challenged with chemotherapeutic agents [9]. However, the molecular mechanisms underlying KIFC1’s role in maintaining spindle stability have not been fully elucidated. For instance, as a transport protein, it remains unidentified as to which proteins KIFC1 transports. Or as a motor protein, it remains ambiguous as to which proteins KIFC1 directly or indirectly binds with, and regulates in terms of their functional attributes.

In this study, we will examine the protein expression of KIFC1 in ESCC in clinical samples. The potential of KIFC1 as a therapeutic target will be evaluated using xenograft models in mice. Through bioinformatics and proteomics, we will further investigate the molecular mechanisms underpinning KIFC1’s role in fostering tumor progression.

## MATERIALS AND METHODS

### ESCC tissue microarray and IHC staining

ESCC tissue microarrays were purchased from Shanghai Outdo Biotech in Shanghai, China (HEsoS060CS01). Experiments using human samples have been approved by the Human Ethics Committee. For immunohistochemistry staining, antibodies against KIFC1(Abcam, ab172620, 1:500) were used. The immunohistochemistry kit was purchased from Sangon Biotech (D601037). For measuring the percentage of positive cells and the intensity of the stain, we quantitatively scored the tissue slides under a microscope. The A.H. score (maximum score 300) was calculated using the formula: 3 × percentage of ultra-stained tissue in comparison to 2 × percentage of moderately stained tissue in comparison to 2% of weakly stained tissue [17]. In a blinded study, two pathologists validated the scoring system independently.

### Cell culture and reagents

EC109 ESCC cell lines were purchased from National Laboratory cell resource sharing platform (Beijing, China). EC109 were recovered and cultured with DMEM (Gibco, Grand Island, NY, USA) medium containing 10% bovine serum (Gibco, Grand Island, NY, USA) until the cells were >80% confluent.

### Clonal formation assay

EC109 cells were initially trypsinized and counted before ten hundred cells were plated in a 6-well plate. The cells were then maintained in a humidified incubator at 37°C and 5% CO_2_ for 10 days until colonies formed. Post formation, the medium was removed, cells were washed with PBS, then fixed with formaldehyde, and subsequently stained with crystal violet (Sangon Biotech, E607309).

### Tumor-bearing mouse models

Mouse related experiments were permitted by the animal ethics committee of Changzhi Medical College, and all experiments were performed in accordance with relevant guidelines and regulations. We confirmed that the mouse experiments were carried out in compliance with the ARRIVE guidelines and approved by the Animal Ethics Committee of Changzhi Medical College. All animals were kept in pathogen-free temperature- and humidity-controlled rooms under a 12-hour light/dark cycle and were provided with soya-free laboratory food and tap water. In female BALB/c nude mice (5 weeks old), EC109 cells were injected subcutaneously into the left flank. Every two days, mouse tumor size and survival were assessed when tumor volumes reached 300 mm^3^, and animals were sacrificed using CO_2_ inhalation.

### Transfection

The EC109 cells were transfected using Lipofectamine 2000 (Invitrogen, 11668500) according to the manufacturer’s protocol for 48 h in 6-well plates. The transfected cells were used for protein extraction or other subsequent assays. siRNA sequence: sense 5′-UAACUGACCCUUUAAGUCCUU-3′. Furthermore, we used this sequence to design a lentiviral vector targeting KIFC1, which was used to screen for stable cell lines with KIFC1 knockdown (Bocui Technology, Beijing, China). Additionally, based on the full-length CDS sequence of KIFC1, we constructed the PCDNA3.1-KIFC1 expression vector for transient overexpression of KIFC1 protein in cells (Bocui technology).

### Bioinformatics analysis

The RNA-sequencing expression profiles (level 3) and corresponding clinical data for ESCC were procured from the TCGA dataset (http://www.cancer.gov/). The limma package within the R software suite was utilized for the identification of differentially expressed mRNAs, with an adjusted *P*-value < 0.05 and Log2 (Fold Change)>1 or Log2 (Fold Change) <−1 set as the threshold for differential expression.

To further elucidate the possible functions of the potential targets, the acquired data underwent functional enrichment analysis. The Gene Ontology (GO) tool, prevalently employed for gene annotation, shed light on molecular function (MF), biological pathways (BP), and cellular components (CC). The ClusterProfiler package (version: 3.18.0) in R contributed to the analysis of the GO function of potential targets for a comprehensive understanding of mRNA’s role in carcinogenesis. The R software’s ggplot2 and heatmap packages were utilized for the creation of boxplots and heatmaps respectively.

The GSVA package in R software was utilized for the analysis, with ‘ssgsea’ selected as the method parameter. The interrelation between genes and pathway scores was explored through the Spearman correlation. All these analytical techniques and R packages were executed utilizing R version 4.0.3. A *p*-value < 0.05 was deemed as an indicator of statistical significance.

### Immunofluorescence staining (IF)

We cultured ESCC cells on coverslips and fixed them in 4% paraformaldehyde after washing three times in 0.1 M PBS for 3 min each. A bovine serum albumin solution was immersed in the sample for 60 minutes at room temperature and then incubated at 4°C overnight with anti-gama-tubulin antibody (Abcam, ab179503, 1:500), anti-KIFC1 antibody (Abcam, ab172620, 1:1000) or anti-Aurora B antibody (Abcam, ab3609, 1:1000). Coverslips were washed 3 times in PBS for 3 minutes each, then incubated for 60 minutes with a secondary antibody. Goat Anti-Rabbit IgG H&L (Alexa Fluor^®^ 488, Abcam, ab150077, 1:1000) and Goat Anti-Mouse IgG H&L (Alexa Fluor^®^ 594, Abcam, ab150116, 1:1000) were used as the secondary antibody for IF staining. Finally, histology mounting medium (Sigma-Aldrich, F6057) was used to mount the slides. Following immunostaining, the cells were examined under a light microscope (Olympus, IX73).

For immunofluorescence staining of tissues, briefly, we first dewaxed and rehydrated the paraffin sections of ESCC tissues. Then, antigen retrieval was performed using tris-EDTA (pH 7.5) solution under high-temperature and high-pressure conditions. After 1 hour of blocking, immunofluorescence staining was conducted using anti-gama-tubulin antibody diluted at a ratio of 1:200, and incubated at 4°C overnight. On the following day, the staining with secondary antibodies was carried out at a dilution ratio of 1:1000. Subsequent steps for mounting and observation were the same as those for cell staining.

### Centrosome counting

For cultured EC109 cells, centrosome counts were derived from three independent experiments, each involving the counting of 300 cells. For ESCC tissue microarrays, centrosome quantification was performed in three distinct non-overlapping regions, with a minimum of 300 cells were valued in each sample.

### Western blot

RIPA (Beyotime, Shanghai, China) lysate was used to extract the total protein from esophageal carcinoma cells. Secondary antibodies raised against rabbit or mouse IgG 1:5000 was incubated at 4°C for 12 hours after washing the membranes six times with TBST for five minutes each. ChemiDOCTM XRS + imager Bio-Rad, and protein levels were quantified using the Olympus Image-Pro Plus software Immunoblots were scanned by the equipment. Antibody used for western blot were as follows: anti-Histone H3 (Abcam, ab1791), anti-S10-Histone H3 (Abcam, ab308372), anti-beta-actin antibody (Abcam, ab8226), Goat Anti-Rabbit IgG H&L (HRP) (Abcam, ab205718), Goat Anti-Mouse IgG H&L (HRP) (Abcam, ab205719). The remaining antibodies have already been described earlier in the text will not be expounded upon further here.

### Protein extraction and digestion

Prepared cells were ground into powder using liquid nitrogen. Approximately 20 mg of powder were resuspended in 200 μL lysis buffer (4% SDS, 100 mM DTT, 150 mM Tris-HCl pH 8.0), followed by quantified with a BCA Protein Assay Kit (Bio-Rad, USA). In brief, the detergent, DTT, and other low-molecular-weight components were removed using UA buffer (8 M Urea, 150 mM Tris-HCl pH 8.0) by repeated ultrafiltration. We then added 100 μL of 0.05 M iodoacetamide to the UA buffer to block reduced cysteine residues, and incubated the samples in darkness for 20 minutes. Following the digestion of the protein suspension with trypsin, peptides were isolated, Nanodrop device (Thermo Fisher Scientific, USA) was used to determine the vacuum-dried peptide concentration. After acidification with 10% trifluoroacetic acid (TFA), the peptides were desalted with a C18 cartridge (Thermo Fisher Scientific, USA).

### LC-MS/MS analysis

Analyses were performed on a Q Exactive HF-X mass spectrometer coupled with a Thermo Fisher Scientific Easy LLC 1200. The phosphopeptides were loaded onto a self-packed column using buffer A for the phosphoproteomic study. At a flow rate of 300 nL/min, peptides were eluted over 110 min using a linear gradient of buffer B from 2–40%. MS scans were acquired from m/z 350 to m/z 1800 with a resolution of 60,000 at m/z 200 and an injection time of 50 ms. Afterwards, data-dependent top 15 MS/MS scans with normalized energy 28 were applied with 15,000 resolution at 200 m/z using higher-energy collision dissociation (HCD). Isolation window size was set to 1.6 Th, and dynamic exclusion duration was 30 seconds.

### Protein-protein docking

To ensure the precision of the docking results, the protein preparation was carried out by utilising AutoDockTools-1.5.7, which involved the manual removal of water molecules from the protein and the addition of polar hydrogen. The GRAMM Docking Web Server was employed for the protein-protein docking process. Subsequently, the derived protein-protein complex was also manually optimised by excluding water and incorporating polar hydrogen through AutoDockTools-1.5.7. Finally, interactions between proteins were forecasted, and a graphic representation of the protein-protein interaction was formulated via PyMOL. The KIFC1(AlphaFold, AF-Q9BW19-F1) protein is depicted as a green cartoon model, Aurora B (PDB, 2BFY) protein is exhibited as a yellow cartoon model and the INCENP (PDB, 2BFY) protein is portrayed as a violet cartoon model, with their respective binding sites demonstrated as corresponding-colored stick structures. Upon concentrating on the binding region, the binding site is represented as a component of the protein to which it is linked.

### Statistics

In this study, student’s *t*-test was used for the analysis between two groups of data, while one-way ANOVA was employed for the analysis of multiple groups of data. The data were presented as mean ± standard deviation.

### Data availability

The datasets used and/or analyzed during the current study are available from the corresponding author on reasonable request.

## RESULTS

### KIFC1 was highly expressed in ESCC and promoted ESCC proliferation

**Figure 1:**
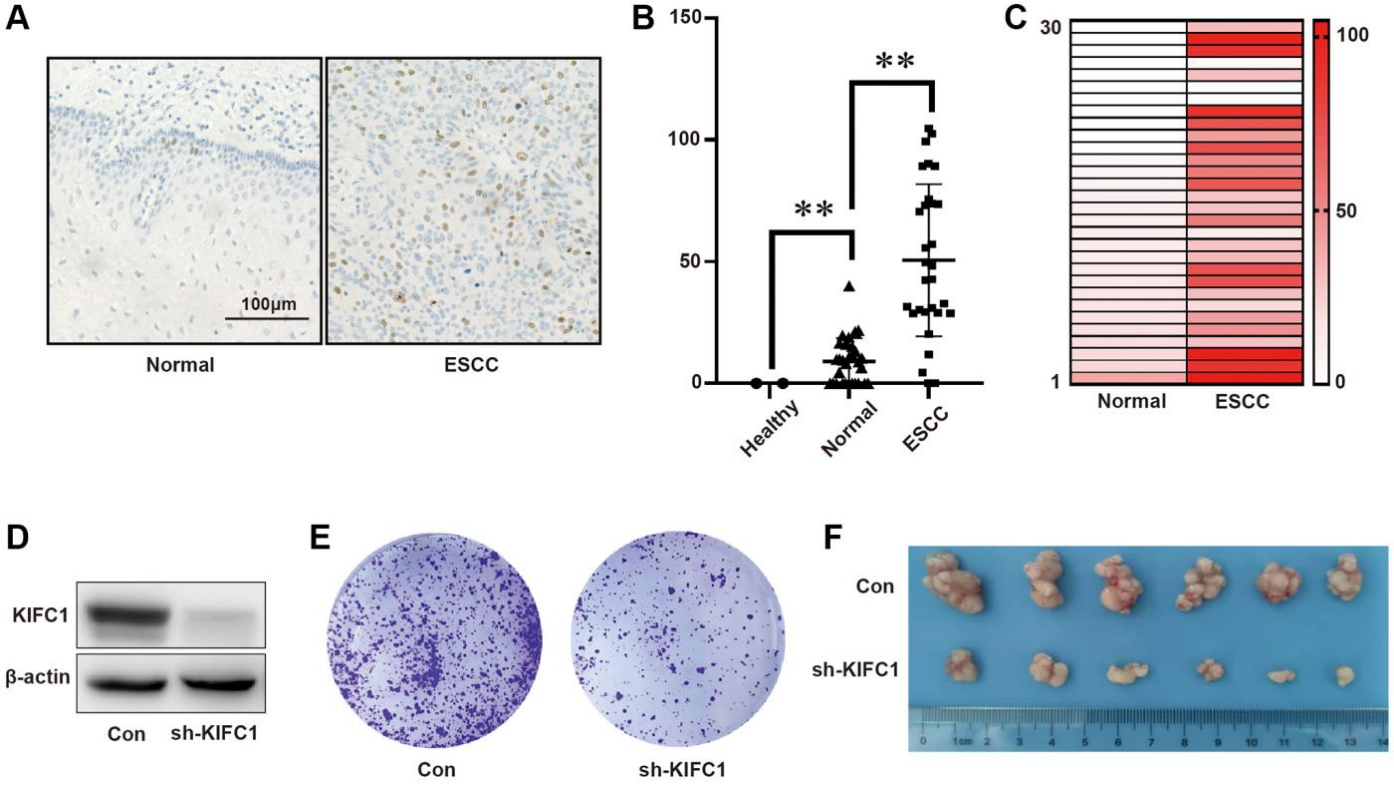
**KIFC1 was overexpressed in tumor tissues and facilitates ESCC Proliferation.** (**A**) Immunohistochemical staining is employed to detect KIFC1 protein expression in both healthy and ESCC tissues; (**B**) The A.H. score is used to statistically analyze KIFC1 protein expression in healthy, adjacent noncancerous, and tumor tissues of the esophagus; (**C**) Analysis of KIFC1 protein expression in adjacent non-tumor esophageal tissues and their paired ESCC tissues is conducted; (**D**) Knock down of KIFC1 using shRNA; (**E**) The influence on clonal formation ability due to knockdown of KIFC1 expression is assessed; (**F**) In vivo analysis of the proliferative ability associated with KIFC1 is performed. *n* = 30, ^**^< 0.01.

KIFC1 exhibits varying prognostic significance in different tumors; however, its role in esophageal cancer remains unreported. Firstly, we investigated the expression of KIFC1 in ESCC and adjacent tissues using an ESCC tissues CHIP (the tissues CHIPs used in this paper was shown in Supplementary Figure 1). As shown in Figure 1A, in the adjacent tissues, KIFC1 was found to be localized in the cell nucleus, with positive cells predominantly present in the basal layer of the esophageal epithelium, albeit with a very low proportion of positive cells. In cancer tissues, the proportion of KIFC1-positive cells increased, although not all cells exhibited positive staining. This could be attributed to the fact that not all tumor cells undergo mitosis in the cancer tissues. Furthermore, we employed the A.H. scoring method (maximum score of 300) to evaluate the expression levels of KIFC1 in healthy tissues, adjacent tissues, and ESCC tissues. The results revealed a significant increase in KIFC1 scores in cancer tissues compared to adjacent tissues. However, there was considerable variation in KIFC1 expression levels within the cancer tissues (Figure 1B). The paired analysis of KIFC1 protein expression was shown in Figure 1C. We significantly knocked down the expression of KIFC1 using shRNA (Figure 1D). In the cancer tissues, the expression level of KIFC1 was significantly higher than that in adjacent non-tumor tissues, though negative expression samples were also present. At the cellular level, through the clone formation experiment, we found that after transfection of EC109 cells with shRNA plasmid targeting KIFC1, the clone formation ability of EC109 cells significantly weakened (Figure 1E). As expected, the tumorigenic ability of EC109 cells with KIFC1 knockdown in nude mice subcutaneously was greatly reduced (Figure 1F). The results indicate that the expression of KIFC1 significantly increases in ESCC tissues, and the knockdown of KIFC1 could greatly reduce the proliferation capability of tumor cells, potentially having significant prognostic value.

### Bioinformatics analysis of signaling pathways associated with KIFC1

**Figure 2:**
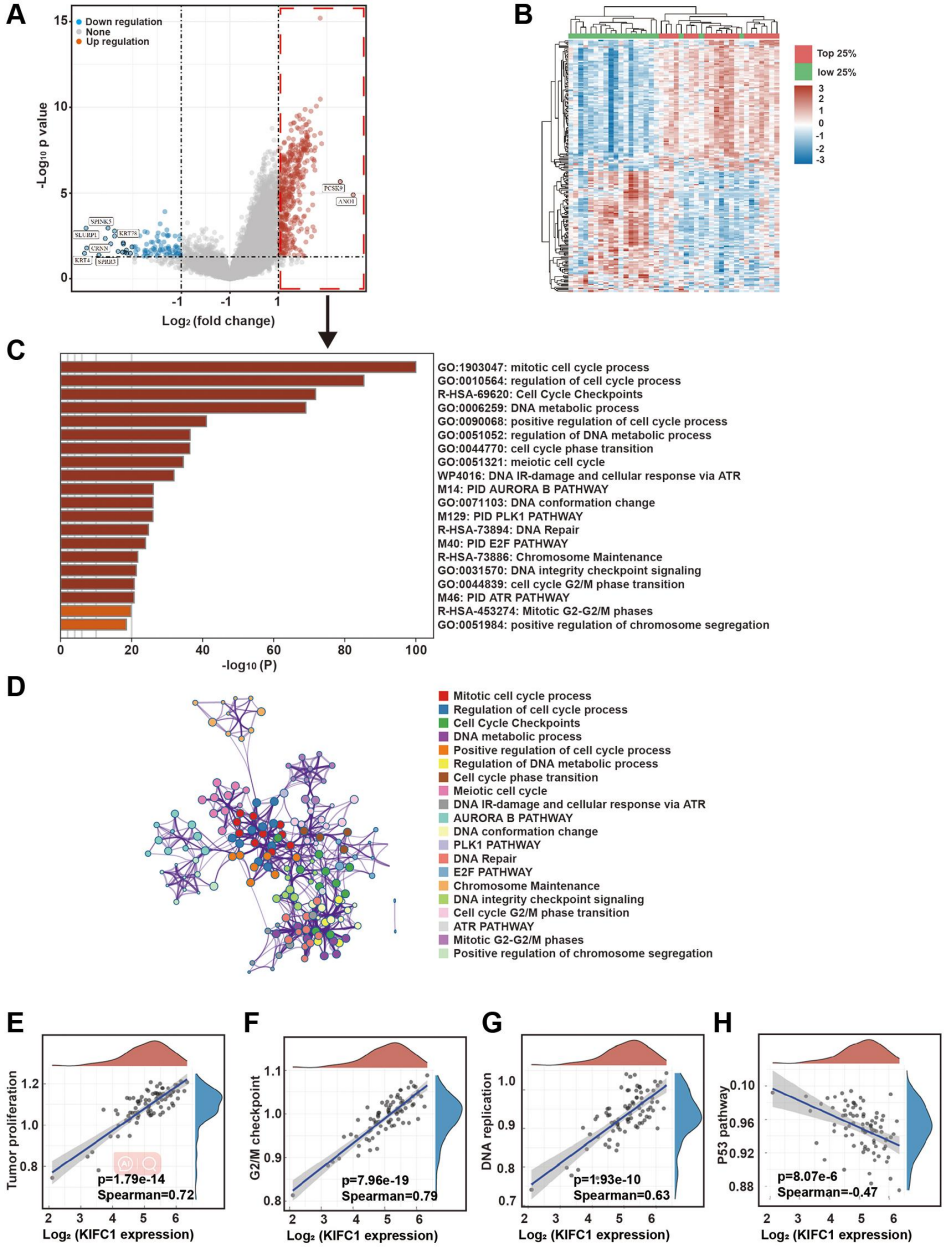
**Bioinformatics analysis of signaling pathways associated with KIFC1.** (**A**) Heatmap of gene expression by grouping TCGA samples based on the level of KIFC1 expression; (**B**) Volcano plot of differential genes between the top 25% of samples with high KIFC1 expression and the bottom 25% of samples with low KIFC1 expression; (**C**) Clustering analysis of upregulated differentially expressed genes in the top 25% of samples with high KIFC1 expression; (**D**) Network of enriched term; (**E–H**) Correlation analysis of KIFC1 expression with tumor proliferation, G2/M checkpoints, DNA replication, and the p53 pathway.

In order to investigate the mechanism through which KIFC1 promotes proliferation in esophageal carcinoma cells, we utilized the data from 82 ESCC cases in TCGA for analysis. The heat map of KIFC1 expression from the bioinformatics analysis was shown in Figure 2A. Further, we divided all samples into 4 groups according to the expression level of KIFC1. We analyzed the differential genes between the top 25% of samples and the bottom 25% of samples based on KIFC1 expression level. The volcano plot of these differential genes was shown in Figure 2B. The GO clustering analysis of differential genes indicates that, compared to samples with low KIFC1 expression, the genes that were elevated in the KIFC1 high expression group were clustered in numerous cell cycle-related pathways (Figure 2C). The network of pathways was shown in Figure 2D. Correlation analysis of these pathways indicates a highly significant positive correlation between KIFC1 expression and tumor proliferation pathways (Figure 2E, Spearman coefficient = 0.72), G2/M checkpoints (Figure 2F, Spearman coefficient = 0.79), and DNA replication pathway (Figure 2G, Spearman coefficient = 0.63). Additionally, there was a highly significant negative correlation with the P53 pathway (Figure 2H, Spearman coefficient = −0.47). The above analysis suggests that high expression of KIFC1 was a sign of vigorous cell division. Elevated expression of KIFC1 could potentially affect the cell’s mitotic process.

### Overexpression of KIFC1 promotes centrosome amplification in EC109 cells

**Figure 3:**
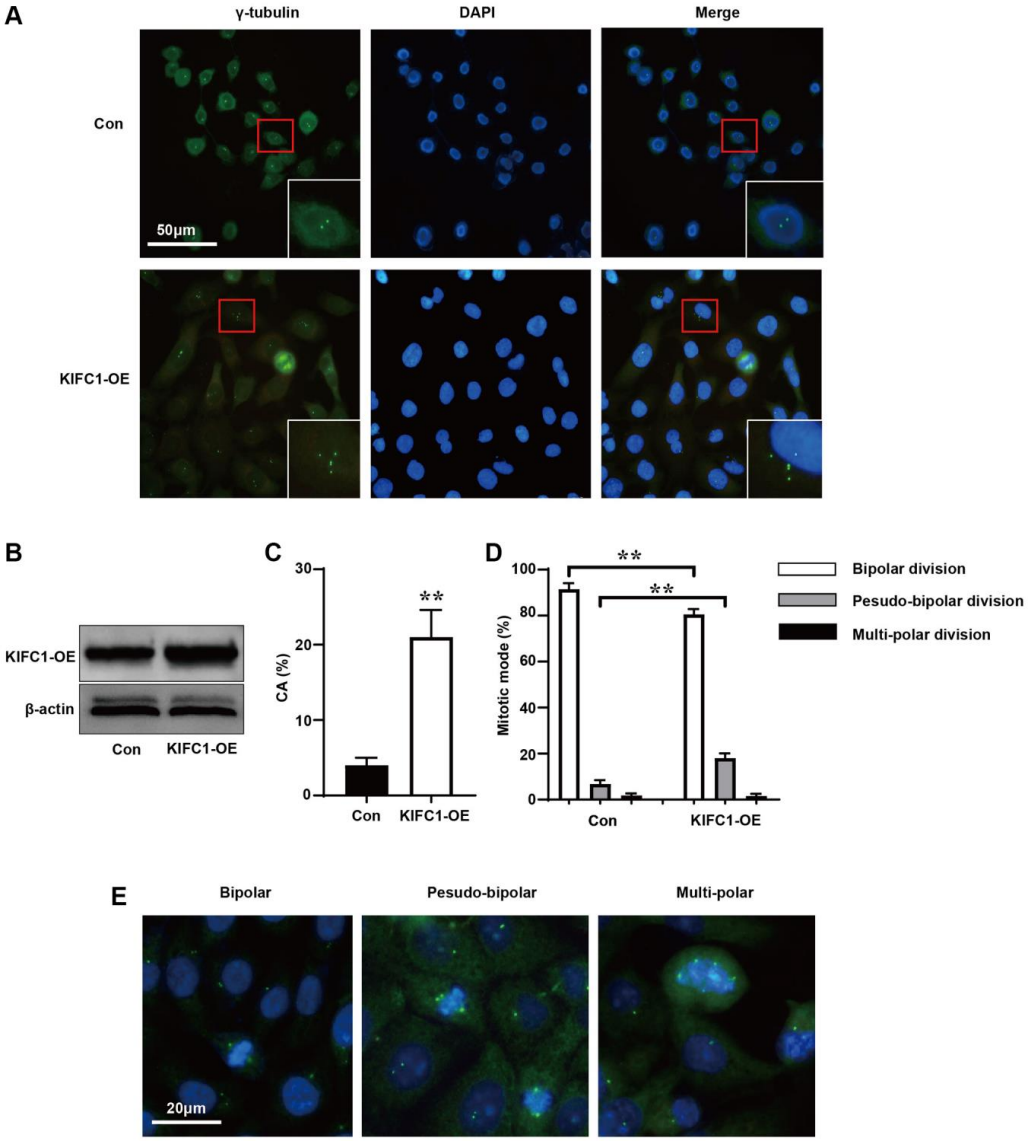
**Overexpression of KIFC1 promotes centrosome amplification and pseudo-bipolar division.** (**A**) Schematic illustration of centrosome amplification in KIFC1 overexpression group and control group, where centrosomes were labeled with gama-tubulin antibody (green) and DNA was stained with DAPI (blue); (**B**) Western blot analysis the overexpression of KIFC1; (**C**) Statistics of centrosome amplification rate in cells after KIFC1 overexpression; (**D**) Statistics of the ways cells enter mid and late stages of mitosis; (**E**) Schematic illustrations of bipolar division, pseudo-bipolar division, and multi-polar division in cells, where centrosomes were labeled with gama-tubulin antibody (green) and DNA was stained with DAPI (blue). *n* = 3, ^**^< 0.01.

To further investigate the relationship between KIFC1 and cell division, we used an anti-gamma-tubulin antibody to label the centrosomes and spindle of control group cells and cells overexpressing KIFC1 (Figure 3A). When KIFC1 were overexpressed (Figure 3B), cells in the KIFC1 overexpression group showed more instances of centrosome amplification (Figure 3C). Further, we statistics the ways in which cells entered the metaphase and anaphase of cell cycle before and after overexpression of KIFC1. Although there were more instances of centrosome amplification in cells overexpressing KIFC1, the cells tended to complete mitosis through pseudo-bipolar division (Figure 3D), in which extra centrosomes tended to cluster at the two poles of the spindle (Figure 3E).

### The expression of KIFC1 and centrosome amplification demonstrate a significant association in the context of ESCC

**Figure 4:**
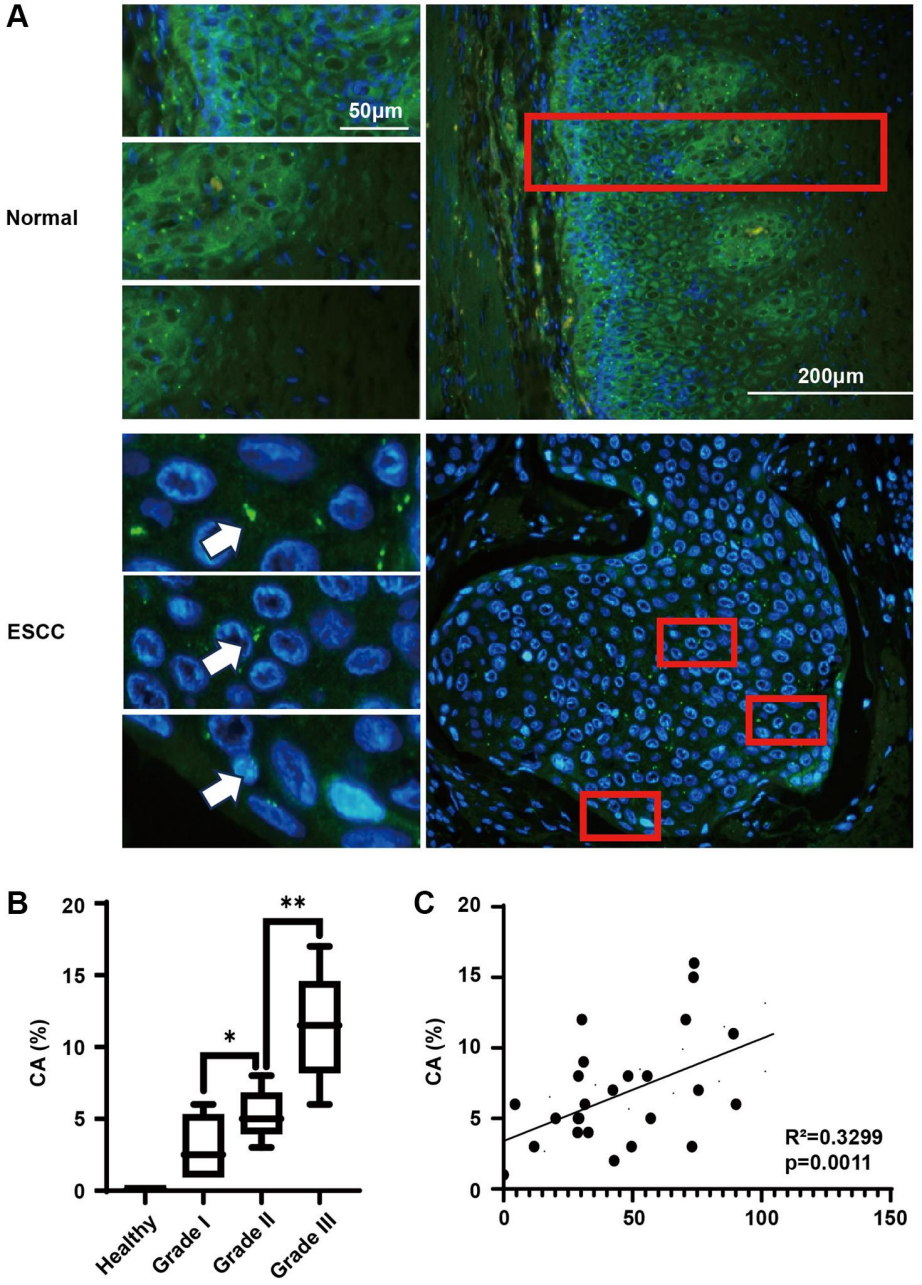
**Centrosome amplification in ESCC tissues and paired analysis with KIFC1 expression levels.** (**A**) Centrosome staining in ESCC tissues and adjacent tissues, centrosomes marked using anti-gamma-tubulin antibodies (green) and DNA stained with DAPI (blue); (**B**) Analysis of centrosome amplification phenomena in relation to tumor grade; (**C**) Analysis of the relationship between KIFC1 expression levels and rates of centrosome amplification. *n* = 30, ^*^< 0.05, ^**^< 0.01.

Presently, at the cellular level, we have identified an association between KIFC1 overexpression and centrosome amplification. We subsequently utilized the same esophageal cancer tissue microarray to conduct immunofluorescence investigations (Figure 4A). In the adjacent cancerous tissues, all cells host 1-2 centrosomes, with basal cells and glandular tube cells exhibiting stronger fluorescence intensities. From the basal to epidermal layers, a gradual decrease in gamma-tubulin staining intensity was observed, likely due to the differentiation of basal cells to squamous epithelium. In ESCC tissues, we frequently observe amplified centrosomes, and the gamma-tubulin staining exhibits a more homogenous intensity. After a comprehensive statistical analysis, we noticed that centrosome amplification was absent in normal esophageal epithelial tissue but was apparent in ESCC tissue, with amplification intensifying in line with elevated tumor grades (Figure 4B). Given that the protein expression level of KIFC1 and the detection of centrosome amplification arise from the same tissue microarray, we paired these two sets of statistical outcomes. The relationship was presented in a scatter diagram (Figure 4C), demonstrating a significant positive correlation between the expression level of KIFC1 and the ratio of centrosome amplification (Spearman’s coefficient = 0.3299).

### Proteomics identification of the proteins interacting with KIFC1

**Figure 5:**
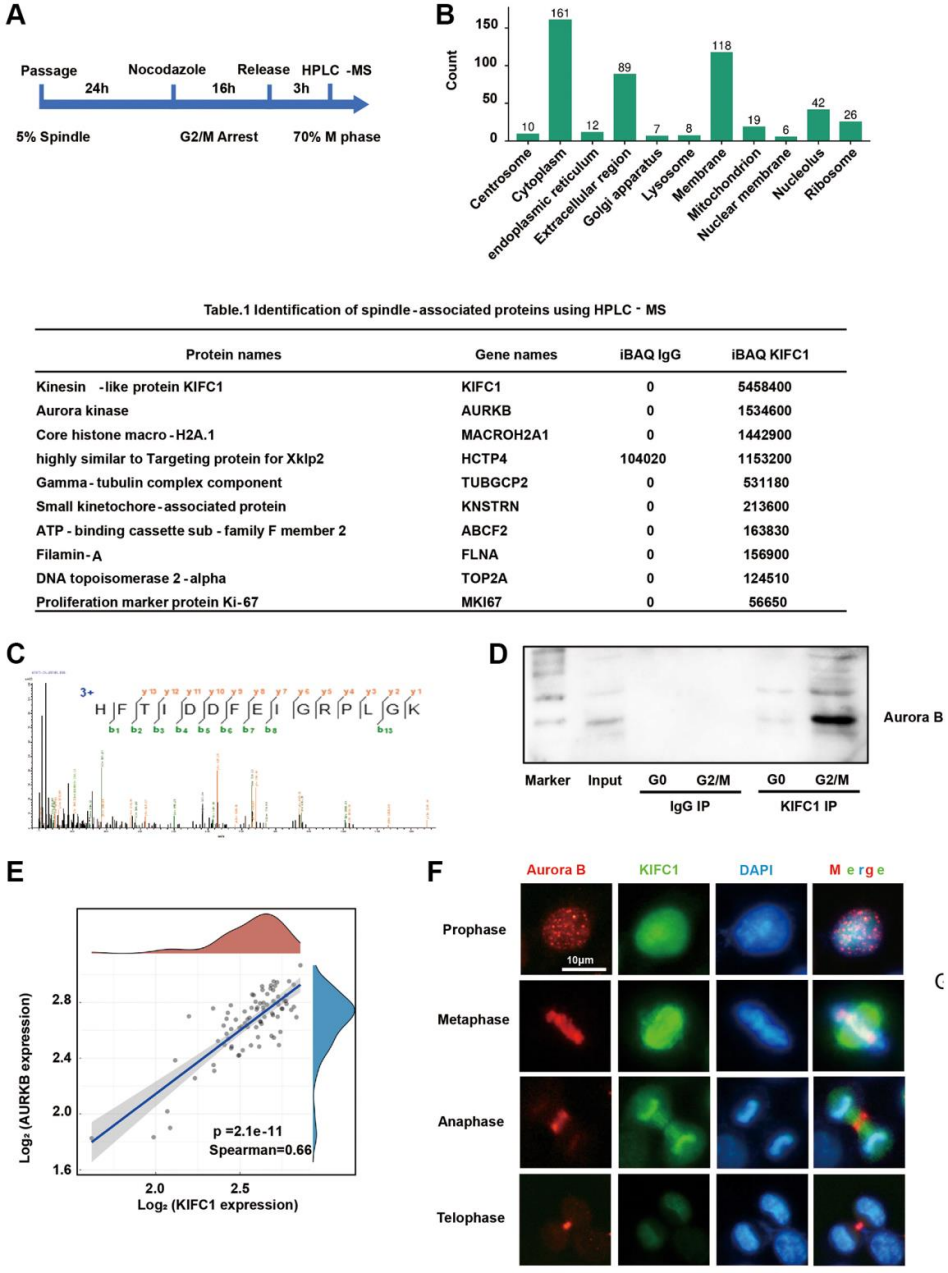
**Proteomic analysis reveals protein interactions with KIFC1.** (**A**) Flow chart of metaphase cell; (**B**) GO cluster of identification results, clustering according to organelles; (**C**) Mass spectrum peak of Aurora B; (**D**) Immunoprecipitation with anti KIFC1 antibody and detect Aurora B content through western blot; (**E**) Bioinformatics analysis of the correlation between KIFC1 and Aurora B mRNA expression; (**F**) immunofluorescence stain of KIFC1 (green) and Aurora B (red), DNA were stained with DAPI (blue).

KIFC1 exhibits dual functions in microtubule organization and vesicle transport, which have not been thoroughly explored. To investigate the molecular mechanisms through which KIFC1 promote centrosome amplification and pseudo-bipolar division, we treated cells with nocodazole to enrich for G2/M phase cells. After release, an estimated 70% of the cells progressed to the metaphase. Co-immunoprecipitation (Co-IP) and high-performance liquid chromatography-mass spectrometry (HPLC-MS) were utilized to identify the proteins interacting either directly or indirectly with KIFC1 (Figure 5A). GO clustering allowed us to focus on proteins localizing to the spindle (Figure 5B). The spindle associate proteins were shown in Table 1. (In Supplementary Table 1, we present all the proteins that directly or indirectly interact with KIFC1, and their interaction network was shown in Supplementary Figure 2). Intriguingly, Aurora B was the most powerful protein amongst all contenders (Figure 5C). Co-IP and western blot experiments revealed a significant rise in the binding between KIFC1 and Aurora B during metaphase (Figure 5D). A bioinformatics analysis demonstrated a substantial positive correlation (Figure 5E, spearman = 0.66) between KIFC1 and Aurora B mRNA expression levels in ESCC. Furthermore, we confirmed the binding of KIFC1 and Aurora B through co-localization using immunofluorescence techniques (Figure 5F).

**Table 1:** Identification of spindle-associated proteins using HPLC-MS.

**Protein names**	**Gene names**	**iBAQ IgG**	**iBAQ KIFC1**
Kinesin-like protein KIFC1	KIFC1	0	5458400
Aurora kinase	AURKB	0	1534600
Core histone macro-H2A.1	MACROH2A1	0	1442900
Highly similar to Targeting protein for Xklp2	HCTP4	104020	1153200
Gamma-tubulin complex component	TUBGCP2	0	531180
Small kinetochore-associated protein	KNSTRN	0	213600
ATP-binding cassette sub-family F member 2	ABCF2	0	163830
Filamin-A	FLNA	0	156900
DNA topoisomerase 2-alpha	TOP2A	0	124510
Proliferation marker protein Ki-67	MKI67	0	56650

### KIFC1 regulates Aurora B protein expression and subcellular localization

**Figure 6:**
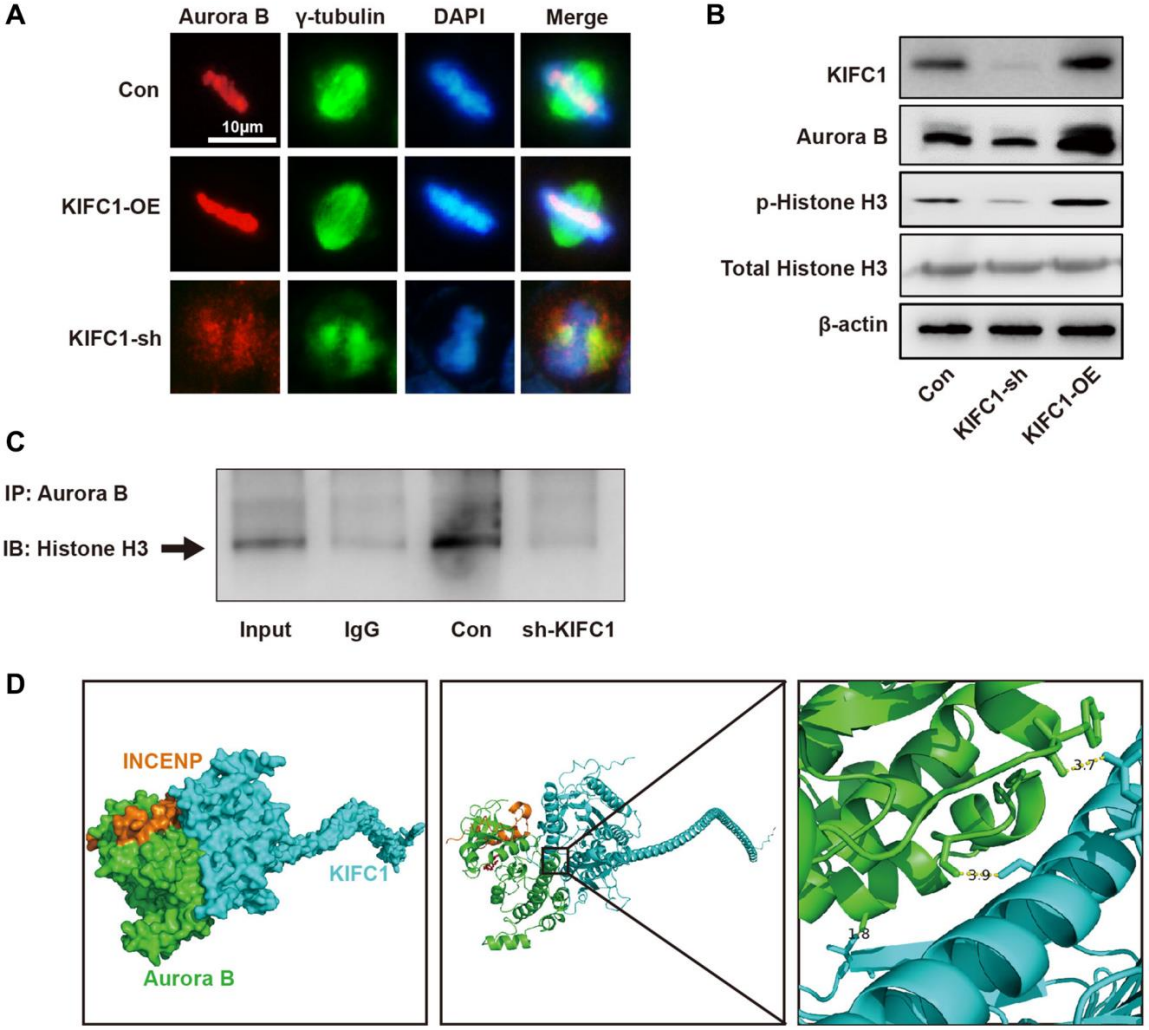
**KIFC1 regulates the expression and subcellular localization of Aurora B.** (**A**) The subcellular localization of Aurora B after overexpression and knockdown of KIFC1, where Aurora B is indicated in red, and gamma tubulin in green; (**B**) Protein levels of Aurora B and the expression and phosphorylation of its substrate Histone H3 after overexpression and knockdown of KIFC1; (**C**) co-IP detect the binding of Aurora B and Histone H3; (**D**) The molecular docking model of KIFC1 with Aurora B-INCENP complex.

To investigate the regulatory mechanisms of KIFC1 on Aurora B, we manipulated KIFC1 expression through both knockdown and overexpression approaches and conducted subsequent studies via co-localization immunofluorescence and western blot. Upon overexpression of KIFC1 within cells, Aurora B displayed proper subcellular localization, and a significant augmentation in fluorescence intensity was observed. However, the ablation of KIFC1 resulted in a mis-localization of Aurora B outside chromosomes accompanied by significant absence in chromosomal location, resulting in abnormal chromosomal morphology (Figure 6A). Examination of protein levels through western blot revealed that KIFC1 knockdown mildly reduced Aurora B protein levels, while overexpression remarkably elevated its levels (Figure 6B). After knocking down KIFC1, the binding between Aurora B and its classical substrate, Histone H3, was significantly reduced (Figure 6C). Lastly, we inspected the protein expression levels and phosphorylation state of Histone H3, a classic Aurora B substrate, and found that the knockdown of KIFC1 significantly reduced the phosphorylation level of Histone H3, with KIFC1 overexpression substantially enhanced it. Finally, we performed molecular docking using the KIFC1 structure from Alpha Fold and the Aurora B- INCENP complex structure listed in the PDB. The docking results indicate that the spatial distances between Aurora B-Tyr860 and KIFC1-Asn617, Aurora b-Ser338 and KIFC1-Ser597, and Aurora b-Ala319 and KIFC1-Thr368 are 3.7, 3.9, and 1.8 angstroms, respectively. There are significant intermolecular hydrophobic interactions present (Figure 6D). These results demonstrate that KIFC1 carries a dual function in regulating the protein levels and subcellular localization of Aurora B, offering a new perspective for understanding the molecular mechanisms by which KIFC1 promotes ESCC cell proliferation and centrosome aberration.

## DISCUSSION

Utilizing immunohistochemical staining of ESCC tissue CHIP, we observed that the expression of KIFC1 was exceptionally low in normal tissues. In contrast, in adjacent tissues, the expression of KIFC1 was significantly increased, while in tumor tissues, the expression of KIFC1 was drastically elevated. This phenomenon might be due to the specific surges in KIFC1 expression in cells during mitosis [10]. Given the substantial difference in KIFC1 expression between normal and ESCC tissues, we considered proteins with similar traits, such as KI67. After comparing the expression levels of KIFC1 and KI67 in ESCC [17] and considering the expression and location of KIFC1 in a normal esophagus, we believe that KIFC1 has the potential to play a significant role in early detection of ESCC.

KIFC1 has dual functions of material transport and microtubule organization. In oocytes deletion of KIFC1 leads to spindle instability and aneuploidy production [4, 5, 18]. The exact mechanism for maintaining spindle stability was also found in cisplatin resistance [9] and neuronal migration [4].

Through bioinformatics analysis, we found that the high expression of KIFC1 is significantly positively correlated with tumor proliferation, DNA replication, and other signaling pathways (Figure 2). Previous research has indicted that the overexpression of KIFC1 could significantly shorten the S phase’s duration and promote the cell cycle’s transition [10]. This suggests us that the increased KIFC1 protein may enhance efficiency of DNA replication and shorten the interphase of mitosis by accelerating protein transport.

The regulation of the cell cycle is characterized by a high degree of precision [19]. During the S phase, cells need to complete the replication of DNA and centrosomes [20]. Nevertheless, an increase in KIFC1 can introduce significant interference in this phase [10], leading us to question the potential effects of KIFC1 on the replication of centrosomes and consequent mitosis. Following immunofluorescence staining results validated our hypothesis, with overexpression of KIFC1 promoting centrosome amplification (Figure 3). Yet, cells exhibit a marked inclination towards pseudo-bipolar division to progress through the cell cycle. This mode of division circumvents the substantial loss of genetic material or mitotic catastrophe caused by multi-polar division, thereby preventing progeny cells from becoming nonviable [21]. Moreover, this method of division could lead to heightened chromosomal instability, thus enhancing tumor heterogeneity [22].

Previous literature has illustrated that KIFC1 localizes to the mitotic spindle [5, 18], connecting adjacent microtubules and preserving spindle stability [23]. However, our observations reveal that follow by the M phase and anaphase, the subcellular localization of KIFC1 does not return to the nucleus, but rather aligns with central spindle (Figure 5F). Studies have shown that the central spindle plays a role in promoting the separation of chromosomes into two daughter cells [24]. However, our understanding of the composition and function of the centrosome is still in its early stages. Currently, we do not know the mechanisms behind its assembly and the proteins that regulate it [25, 26], further research is needed. Using co-immunoprecipitation (Co-IP) with cells disrupted physically rather than by detergents (NP40), we identified numerous candidate proteins transported by KIFC1, including TPX4 (Table 1), through proteomics analysis. Similar as KIFC1, TPX2 (HCTP4) exhibits spindle localization [27], and as prototypical protein playing crucial roles in the cell cycle, it can accelerate the cell cycle by phosphorylating substrate proteins such as Aurora A [28]. TPX2 might directly bind to KIFC1, facilitating phosphorylation of KIFC1, thereby maintain spindle stability. However, this interaction does not suffice to bring centrosomes closer and maintain pseudo-bipolar division.

Among the identified candidates, kinase Aurora B, a spindle assembly checkpoint protein [29], draws our attention. During the intermediate stage of mitosis, Aurora B localizes to kinetochores, and during the telophase, Aurora B could be observed in the cytokinetic ring [30]. At both stages, the localization of Aurora B overlaps with that of KIFC1. Some research indicates the inhibition of Aurora B results in a multi-polar division phenomenon [31], emphasizing the significant role in monitoring pseudo-bipolar division. The interaction between KIFC1 and Aurora B was validated through immunofluorescence and Co-IP+WB experiments. (Figure 5D, 5F).

Subsequently, after knocking down the expression of KIFC1, Aurora B was unable to accurately localize to the metaphase plate during the M phase of mitosis (Figure 6A). It provides evidence to KIFC1’s ability to transport Aurora B to the precise location for its functional execution. The differential dephosphorylation modification of Histone H3, a classical substrate of Aurora B [32], provides empirical evidence for this assertion (Figure 6).

In the progeny cells created by pseudo-bipolar division, the abnormal number of centrosomes inevitably leads to centrosomes amplification. It may cause further instability of genetic materials in cancer cells [33]. Thus, centrosome amplification was considered an important indicator for tumor grading, typing, and prognosis [34, 35]. The overexpression of KIFC1 in cells could significantly elevate the rate of centrosome amplification *in vitro* (Figure 3). Upon staining centrosomes with the same clinical samples used in KIFC1 immunohistochemical experiments, we observed a significant positive correlation between the expression of KIFC1 and the rate of centrosome amplification in ESCC (Figure 4). This is the inaugural research correlating the expression of a protein with centrosome amplification. Previous research on various tumors, including esophageal adenocarcinoma, concluded that the rate of centrosome amplification rises as tumor grade escalates [36], and our study corroborates this. Our findings suggest that KIFC1 might serve as a vital marker for tumorigenic heterogeneity. However, further exploration is warranted to solidify this hypothesis.

In conclusion, our work shows the potential utility of KIFC1 as both a tumor marker and a promising target for therapeutic interventions.

## Supplementary Materials

Supplementary Figures

Supplementary Table 1

## References

[1] Bray F, Ferlay J, Soerjomataram I, Siegel RL, Torre LA, Jemal A. Global cancer statistics 2018: GLOBOCAN estimates of incidence and mortality worldwide for 36 cancers in 185 countries. CA Cancer J Clin. 2018; 68:394–424. 10.3322/caac.21492 30207593

[2] Lin Y, Totsuka Y, Shan B, Wang C, Wei W, Qiao Y, Kikuchi S, Inoue M, Tanaka H, He Y. Esophageal cancer in high-risk areas of China: research progress and challenges. Ann Epidemiol. 2017; 27:215–21. 10.1016/j.annepidem.2016.11.004 28007352

[3] Thrift AP. Global burden and epidemiology of Barrett oesophagus and oesophageal cancer. Nat Rev Gastroenterol Hepatol. 2021; 18:432–43. 10.1038/s41575-021-00419-3 33603224

[4] Muralidharan H, Guha S, Madugula K, Patil A, Bennison SA, Sun X, Toyo-Oka K, Baas PW. KIFC1 Regulates the Trajectory of Neuronal Migration. J Neurosci. 2022; 42:2149–65. 10.1523/JNEUROSCI.1708-21.2022 35046122 PMC8936618

[5] Shan MM, Zou YJ, Pan ZN, Zhang HL, Xu Y, Ju JQ, Sun SC. Kinesin motor KIFC1 is required for tubulin acetylation and actin-dependent spindle migration in mouse oocyte meiosis. Development. 2022; 149:dev200231. 10.1242/dev.200231 35142352

[6] Sekino Y, Pham QT, Kobatake K, Kitano H, Ikeda K, Goto K, Hayashi T, Nakahara H, Sentani K, Oue N, Yasui W, Teishima J, Hinata N. KIFC1 Is Associated with Basal Type, Cisplatin Resistance, PD-L1 Expression and Poor Prognosis in Bladder Cancer. J Clin Med. 2021; 10:4837. 10.3390/jcm10214837 34768355 PMC8584707

[7] Wright N, Gong Z, Kittles R, Natarajan R, Jovanovic-Talisman T, Rida P, LaBarge M, Seewaldt V. Kinesin Family Member C1 (KIFC1/HSET): A Potential Actionable Biomarker of Early Stage Breast Tumorigenesis and Progression of High-Risk Lesions. J Pers Med. 2021; 11:1361. 10.3390/jpm11121361 34945833 PMC8708236

[8] Pannu V, Rida PC, Ogden A, Turaga RC, Donthamsetty S, Bowen NJ, Rudd K, Gupta MV, Reid MD, Cantuaria G, Walczak CE, Aneja R. HSET overexpression fuels tumor progression via centrosome clustering-independent mechanisms in breast cancer patients. Oncotarget. 2015; 6:6076–91. 10.18632/oncotarget.3475 25788277 PMC4467423

[9] Fan G, Sun L, Meng L, Hu C, Wang X, Shi Z, Hu C, Han Y, Yang Q, Cao L, Zhang X, Zhang Y, Song X, et al. The ATM and ATR kinases regulate centrosome clustering and tumor recurrence by targeting KIFC1 phosphorylation. Nat Commun. 2021; 12:20. 10.1038/s41467-020-20208-x 33397932 PMC7782532

[10] Wei YL, Yang WX. Kinesin-14 motor protein KIFC1 participates in DNA synthesis and chromatin maintenance. Cell Death Dis. 2019; 10:402. 10.1038/s41419-019-1619-9 31127080 PMC6534603

[11] Zhou K, Lin J, Dai M, He Y, Xu J, Lin Q. KIFC1 promotes aerobic glycolysis in endometrial cancer cells by regulating the c-myc pathway. J Bioenerg Biomembr. 2021; 53:703–13. 10.1007/s10863-021-09924-1 34729671

[12] Muralidharan H, Baas PW. Mitotic Motor KIFC1 Is an Organizer of Microtubules in the Axon. J Neurosci. 2019; 39:3792–811. 10.1523/JNEUROSCI.3099-18.2019 30804089 PMC6520510

[13] Teng K, Wei S, Zhang C, Chen J, Chen J, Xiao K, Liu J, Dai M, Guan X, Yun J, Xie D. KIFC1 is activated by TCF-4 and promotes hepatocellular carcinoma pathogenesis by regulating HMGA1 transcriptional activity. J Exp Clin Cancer Res. 2019; 38:329. 10.1186/s13046-019-1331-8 31340839 PMC6657086

[14] Han J, Wang F, Lan Y, Wang J, Nie C, Liang Y, Song R, Zheng T, Pan S, Pei T, Xie C, Yang G, Liu X, et al. KIFC1 regulated by miR-532-3p promotes epithelial-to-mesenchymal transition and metastasis of hepatocellular carcinoma via gankyrin/AKT signaling. Oncogene. 2019; 38:406–20. 10.1038/s41388-018-0440-8 30115976 PMC6336682

[15] Li J, Diao H, Guan X, Tian X. Kinesin Family Member C1 (KIFC1) Regulated by Centrosome Protein E (CENPE) Promotes Proliferation, Migration, and Epithelial-Mesenchymal Transition of Ovarian Cancer. Med Sci Monit. 2020; 26:e927869. 10.12659/MSM.927869 33361741 PMC7780892

[16] Akabane S, Oue N, Sekino Y, Asai R, Thang PQ, Taniyama D, Sentani K, Yukawa M, Toda T, Kimura KI, Egi H, Shimizu W, Ohdan H, Yasui W. KIFC1 regulates ZWINT to promote tumor progression and spheroid formation in colorectal cancer. Pathol Int. 2021; 71:441–52. 10.1111/pin.13098 33819373

[17] Sjöstedt E, Zhong W, Fagerberg L, Karlsson M, Mitsios N, Adori C, Oksvold P, Edfors F, Limiszewska A, Hikmet F, Huang J, Du Y, Lin L, et al. An atlas of the protein-coding genes in the human, pig, and mouse brain. Science. 2020; 367:eaay5947. 10.1126/science.aay5947 32139519

[18] So C, Menelaou K, Uraji J, Harasimov K, Steyer AM, Seres KB, Bucevičius J, Lukinavičius G, Möbius W, Sibold C, Tandler-Schneider A, Eckel H, Moltrecht R, et al. Mechanism of spindle pole organization and instability in human oocytes. Science. 2022; 375:eabj3944. 10.1126/science.abj3944 35143306

[19] Liu J, Peng Y, Wei W. Cell cycle on the crossroad of tumorigenesis and cancer therapy. Trends Cell Biol. 2022; 32:30–44. 10.1016/j.tcb.2021.07.001 34304958 PMC8688170

[20] Prigent C, Uzbekov R. Duplication and Segregation of Centrosomes during Cell Division. Cells. 2022; 11:2445. 10.3390/cells11152445 35954289 PMC9367774

[21] Milunović-Jevtić A, Mooney P, Sulerud T, Bisht J, Gatlin JC. Centrosomal clustering contributes to chromosomal instability and cancer. Curr Opin Biotechnol. 2016; 40:113–8. 10.1016/j.copbio.2016.03.011 27046071 PMC4975617

[22] Cosenza MR, Cazzola A, Rossberg A, Schieber NL, Konotop G, Bausch E, Slynko A, Holland-Letz T, Raab MS, Dubash T, Glimm H, Poppelreuther S, Herold-Mende C, et al. Asymmetric Centriole Numbers at Spindle Poles Cause Chromosome Missegregation in Cancer. Cell Rep. 2017; 20:1906–20. 10.1016/j.celrep.2017.08.005 28834753

[23] Sun M, Jia M, Ren H, Yang B, Chi W, Xin G, Jiang Q, Zhang C. NuMA regulates mitotic spindle assembly, structural dynamics and function via phase separation. Nat Commun. 2021; 12:7157. 10.1038/s41467-021-27528-6 34887424 PMC8660824

[24] Yu CH, Redemann S, Wu HY, Kiewisz R, Yoo TY, Conway W, Farhadifar R, Müller-Reichert T, Needleman D. Central-spindle microtubules are strongly coupled to chromosomes during both anaphase A and anaphase B. Mol Biol Cell. 2019; 30:2503–14. 10.1091/mbc.E19-01-0074 31339442 PMC6743361

[25] Henson JH, Buckley MW, Yeterian M, Weeks RM, Simerly CR, Shuster CB. Central Spindle Self-Organization and Cytokinesis in Artificially Activated Sea Urchin Eggs. Biol Bull. 2016; 230:85–95. 10.1086/BBLv230n2p85 27132131

[26] Courthéoux T, Reboutier D, Vazeille T, Cremet JY, Benaud C, Vernos I, Prigent C. Microtubule nucleation during central spindle assembly requires NEDD1 phosphorylation on serine 405 by Aurora A. J Cell Sci. 2019; 132:jcs231118. 10.1242/jcs.231118 31028180

[27] King MR, Petry S. Phase separation of TPX2 enhances and spatially coordinates microtubule nucleation. Nat Commun. 2020; 11:270. 10.1038/s41467-019-14087-0 31937751 PMC6959270

[28] Polverino F, Naso FD, Asteriti IA, Palmerini V, Singh D, Valente D, Bird AW, Rosa A, Mapelli M, Guarguaglini G. The Aurora-A/TPX2 Axis Directs Spindle Orientation in Adherent Human Cells by Regulating NuMA and Microtubule Stability. Curr Biol. 2021; 31:658–67.e5. 10.1016/j.cub.2020.10.096 33275894

[29] Borisa A, Bhatt H. 3D-QSAR (CoMFA, CoMFA-RG, CoMSIA) and molecular docking study of thienopyrimidine and thienopyridine derivatives to explore structural requirements for aurora-B kinase inhibition. Eur J Pharm Sci. 2015; 79:1–12. 10.1016/j.ejps.2015.08.017 26343315

[30] Kurasawa Y, Lee KJ, Hu H, Pham KTM, Li Z. Polo-like kinase and Aurora B kinase phosphorylate and cooperate with the CIF1-CIF2 complex to promote cytokinesis initiation in *Trypanosoma brucei*. Open Biol. 2022; 12:220197. 10.1098/rsob.220197 36196534 PMC9532997

[31] Yan Z, Shi Q, Liu X, Li J, Ahire V, Zhang S, Zhang J, Yang D, Allen TD. The phytochemical, corynoline, diminishes Aurora kinase B activity to induce mitotic defect and polyploidy. Biomed Pharmacother. 2022; 147:112645. 10.1016/j.biopha.2022.112645 35051862

[32] Hirota T, Lipp JJ, Toh BH, Peters JM. Histone H3 serine 10 phosphorylation by Aurora B causes HP1 dissociation from heterochromatin. Nature. 2005; 438:1176–80. 10.1038/nature04254 16222244

[33] Mittal K, Kaur J, Jaczko M, Wei G, Toss MS, Rakha EA, Janssen EAM, Søiland H, Kucuk O, Reid MD, Gupta MV, Aneja R. Centrosome amplification: a quantifiable cancer cell trait with prognostic value in solid malignancies. Cancer Metastasis Rev. 2021; 40:319–39. 10.1007/s10555-020-09937-z 33106971 PMC7897259

[34] Yamamoto Y, Misumi T, Eguchi S, Chochi Y, Kitahara S, Nakao M, Nagao K, Hara T, Sakano S, Furuya T, Oga A, Kawauchi S, Sasaki K, Matsuyama H. Centrosome amplification as a putative prognostic biomarker for the classification of urothelial carcinomas. Hum Pathol. 2011; 42:1923–30. 10.1016/j.humpath.2011.02.013 21683985

[35] Denu RA, Zasadil LM, Kanugh C, Laffin J, Weaver BA, Burkard ME. Centrosome amplification induces high grade features and is prognostic of worse outcomes in breast cancer. BMC Cancer. 2016; 16:47. 10.1186/s12885-016-2083-x 26832928 PMC4734858

[36] Lopes CAM, Mesquita M, Cunha AI, Cardoso J, Carapeta S, Laranjeira C, Pinto AE, Pereira-Leal JB, Dias-Pereira A, Bettencourt-Dias M, Chaves P. Centrosome amplification arises before neoplasia and increases upon p53 loss in tumorigenesis. J Cell Biol. 2018; 217:2353–63. 10.1083/jcb.201711191 29739803 PMC6028540

