# Correction for: KIFC1 promotes proliferation and pseudo-bipolar division of ESCC through the transportation of Aurora B kinase

**DOI:** 10.18632/aging.205719

**Published:** 2024-03-31

**Authors:** Bin Du, Lingyu Wei, Jia Wang, Yanyan Li, Jing Huo, Jinsheng Wang, Pu Wang

**Affiliations:** 1 Center of Healthy Aging, Changzhi Medical College, Changzhi 047500, China; 2 Department of Pathology, Affiliated HePing Hospital of Changzhi Medical College, Changzhi 047500, China; 3 Department of Pathology, The First Clinical College of Changzhi Medical College, Changzhi 047500, China; 4 Department of Biology, Changzhi Medical College, Changzhi 047500, China

**Keywords:** KIFC1, Aurora B, ESCC, proliferation, pseudo-bipolar division

**This article has been corrected:** The authors corrected affiliations for the authors Jing Huo^3^ and Jinsheng Wang^4^. The correct authors list with the corresponding affiliations is presented below.

Bin Du^1,*^, Lingyu Wei^2,*^, Jia Wang^1^, Yanyan Li^1^, Jing Huo^3^, Jinsheng Wang^4^, Pu Wang^1^

^1^Center of Healthy Aging, Changzhi Medical College, Changzhi 047500, China

^2^Department of Pathology, Affiliated HePing Hospital of Changzhi Medical College, Changzhi 047500, China

^3^Department of Biology, Changzhi Medical College, Changzhi 047500, China

^4^Department of Pathology, The First Clinical College of Changzhi Medical College, Changzhi 047500, China

^*^Equal contribution and co-first authors

