# Supplementary material for: KIFC1 promotes proliferation and pseudo-bipolar division of ESCC through the transportation of Aurora B kinase
Source: Aging (Albany NY). 2023 Nov 8;15(21):12633–50. doi: 10.18632/aging.205203 (PMC10683620; doi:10.18632/aging.205203)
Supplement: Supplementary Figures [file aging-15-205203-s001.pdf]

SUPPLEMENTARY FIGURES

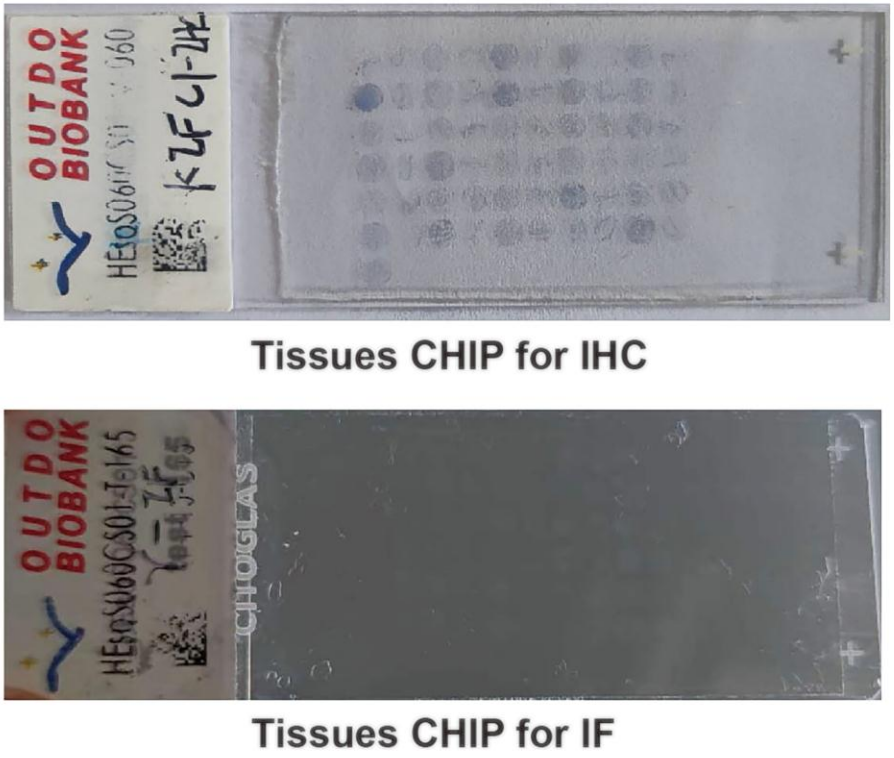

Supplementary Figure 1. Tissues CHIP used in this paper.

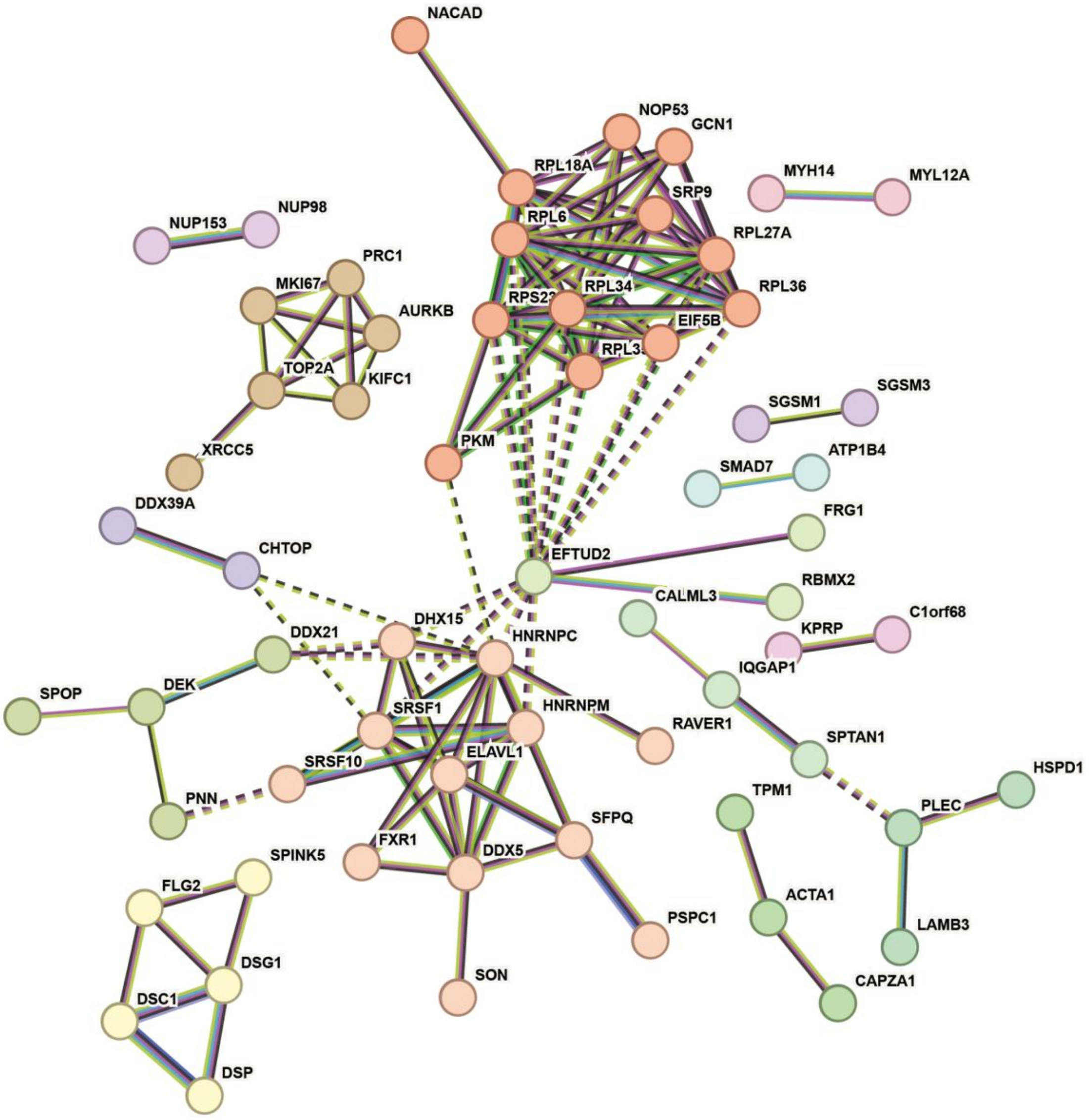

Supplementary Figure 2. Protein interaction network analysis of KIFC1 binding proteins.
